# Effect of administration sequence of induction agents on first‐attempt failure during emergency intubation: A Bayesian analysis of a prospective cohort

**DOI:** 10.1111/acem.15031

**Published:** 2024-10-18

**Authors:** Pierre Catoire, Brian Driver, Matthew E. Prekker, Yonathan Freund

**Affiliations:** ^1^ Emergency Department, Hôpital Pitié‐Salpêtrière Assistance‐Publique Hôpitaux de Paris Paris France; ^2^ Improving Emergency Care (IMPEC) FHU Sorbonne Université Paris France; ^3^ Department of Emergency Medicine Hennepin County Medical Center Minneapolis Minnesota USA; ^4^ Division of Pulmonary, Allergy, and Critical Care Medicine Hennepin County Medical Center Minneapolis Minnesota USA

**Keywords:** Bayesian analysis, drug order, drug sequence, emergency department, intubation, rapid sequence induction, rapid sequence intubation, retrospective study

## Abstract

**Background:**

Emergency tracheal intubation is associated with a risk of clinical adverse events, including the risk of first‐attempt failure. Induction agents usually include a sedative and a neuromuscular blocking agent (i.e., paralytic). Whether the order of administration (i.e., sedative vs. paralytic given first) is associated with first‐attempt failure or adverse events is unknown.

**Methods:**

This study analyzed data from a single‐center prospective cohort collected from 2021 to 2024 at Hennepin County Medical Center, which included all patients undergoing orotracheal intubation in the emergency department. Patients with no detail on administration sequence order were excluded. A Bayesian logistic regression analysis was used to measure the effect of drug sequence order (sedative first vs. paralytic first). The primary outcome was first‐attempt failure. The key secondary outcome was peri‐intubation hypoxemia (SpO_2_ < 90%). We estimated the odds ratio (OR), 95% credible interval (CrI), and the probability that the OR was inferior to 1 (existence of an effect) and inferior to 0.9 (significant effect). Frequentist analysis and reanalysis with various priors were performed as sensitivity analyses.

**Results:**

A total of 2216 patients were included for analysis. The most frequently used sedative and paralytic agents were etomidate (88.9%) and rocuronium (77.8%), respectively. The paralytic was given first to 56.6% of the patients. After adjustment for age, sex, body mass index, and sedative and paralytic agents, the OR for a paralytic‐first strategy for first‐attempt failure was 0.73 (95% CrI 0.46–1.02). The probability that the OR was less than 1 was estimated at 95.7% and less than 0.9 at 87.6%. There was a 33.5% and 8.0% probability that administering the paralytic first resulted in an OR < 1 and OR < 0.9 for the risk of hypoxemia, respectively. Sensitivity analyses were consistent with the main results.

**Conclusions:**

In this Bayesian analysis a paralytic‐first drug sequence was associated with reduced first‐attempt failure during emergency tracheal intubation.

## BACKGROUND

Tracheal intubation in the emergency department (ED) is a common yet high‐risk procedure, with a first‐attempt failure rate of 15% to 20% and a hypoxemia rate (SpO_2_ < 90%) of 10% to 12%.^1^, ^2^, ^3^, ^4^ Rapid‐sequence intubation (RSI), utilizing a near‐simultaneous combination of a sedative and a neuromuscular blockade agent (NMBA, also known as a paralytic), reduces these risks compared to either sedative‐only induction or no induction.^5^ Prior research has compared different sedatives and paralytics, but the effect of the sequence in which these drugs are administered—sedative‐first versus paralytic‐first—remains underexplored. Notably, no consensus exists in international guidelines regarding the preferred sequence, leading to considerable variation in clinical practice; for instance, some high‐performing institutions in the United States administer the paralytic first more than 50% of time,^6^ compared to less than 10% in France.^7^

Sedatives like etomidate and ketamine, at doses commonly used in RSI, have a shorter time to peak clinical effect than most paralytics, suggesting a potential advantage for the sedative‐first strategy by minimizing the risk of paralysis while the patient is awake. However, recent data show no significant increase in awareness of paralysis with a paralytic‐first approach.^8^ Conversely, administering the paralytic first may align the peak clinical effects of the sedative and paralytic agents more closely. This may reduce the duration of apnea caused by the sedative that can occur before the patient is optimized for laryngoscopy and intubation (i.e., paralyzed). Aligning the onset of action may decrease the overall procedure time and risk of peri‐procedure hypoxemia—a principle studied in the operating room setting, but not yet confirmed in the ED context.^9^, ^10^ This study aims to address this gap by assessing the effects of drug sequence order on first attempt failure, peri‐intubation hypoxemia, and complications of emergency tracheal intubation.

## METHODS

### Aim of the study and outcomes

The study's main objective was to assess the association of drug sequence order, comparing sedative‐first and paralytic‐first strategies, with the risk of first‐attempt failure during emergency tracheal intubation. The secondary objectives were to measure the association with peri‐procedure hypoxemia, and occurrence of a major or any complication.

### Study design and setting

The cohort was constituted from January 2021 through January 2024 at Hennepin County Medical Center, through systematic prospective consecutive collection of data from all patients undergoing tracheal intubation in the emergency department (ED). The study hospital is an urban Level‐I adult and pediatric trauma center in the United States, with more than 100,000 annual ED visits and 1000 to 1500 intubations performed annually. Senior resident physicians perform more than 95% of intubation procedures.^11^ Emergency physicians have sole decision making and procedural responsibility for tracheal intubation in the ED. In particular, drug selection, dose and administration sequence order were decided based on physician's preferences and patient characteristics. The present study was designed after data collection. The study complies with the Strengthening the Reporting of Observational Studies in Epidemiology (STROBE) statement.^12^ Use of these data were approved by the local institutional review board.

### Selection of patients

Data on all patients undergoing orotracheal intubation in the ED were prospectively collected in the initial database, which is used primarily for quality improvement. Patients with missing data on drug sequence order, with acute encephalopathy leading to paralytic‐only induction, with use of intramuscular route, or using any other induction agent than etomidate, ketamine, succinylcholine or rocuronium were excluded. The latter exclusion criteria was justified by the differences in pharmacokinetic characteristics and the small sample size of patients receiving other agents, which could imply heterogeneity of the studied effect.

### Data collection

Trained non‐clinical staff prospectively collected patient demographics, vital signs at induction, induction medications, oxygen saturation during and after the intubation procedure, and first‐attempt success or failure. These staff are mostly undergraduate students, with a day and night presence all week long (except holidays). After the procedure, the intubating physician completed a collection form reporting indication for intubation and occurrence of complications.

### Definition of outcomes

First‐attempt failure was defined as the need to perform more than one insertion of the laryngoscope to complete intubation. Hypoxemia was defined as a pulse oximetry lower than 90% occurring from induction to 1 min after the completion of intubation. Major complication was defined as any of hypoxemia, witnessed aspiration, incident cardiac arrest, esophageal intubation, or first attempt failure. Any complication was defined as any of these major complications, or any of pharyngeal, laryngeal, tracheal or dental trauma, or pneumothorax. Both composite outcomes were defined by the authors to differentiate life‐threatening complications from minor events. To our knowledge, no consensus on definition of major complications is available, and literature is heterogeneous.^13^, ^14^, ^15^

### Statistical methods

A Bayesian approach was used to measure the presence and quantify the magnitude of the hypothesized effect. As an alternative framework to frequentist analysis, Bayesian logistic regression enables the measurement of the probability distribution of a parameter (here, the odds ratio [OR] of drug sequence order for the occurrence of each outcome), given prior information and the observed data and adjusted for confounding factors. This enables measuring the probability that the parameter is superior or inferior to any fixed value. In this instance, the probability that the drug sequence order had an effect on the considered outcome (i.e. OR < 1) was measured. Additionally, to assess the importance of this potential effect, we also estimated the probability of an OR < 0.9, indicating clinically relevant effect.

Selection of relevant covariates was performed using backward‐stepwise method, excluding at each step the covariate with the less significant effect. Candidate covariates included age, sex, body mass index (BMI), heart rate, systolic blood pressure, pulse oximetry at induction, induction agents, and medical versus trauma indication for intubation. Covariates were excluded if their *p*‐value was superior to 0.2. Because BMI is a known risk factor for difficult intubation, it was preserved in the model even if its *p*‐value was superior to 0.2. Heterogeneity of the potential effect depending on induction agents was assessed by measuring the proportion of first‐attempt failure depending on induction sequence order and sedative and paralytic agents.

To preserve parsimony of the model, weakly informative priors (i.e., prior distributions with almost equal likelihood on a wide parameter space) were used through normal distribution, with mean of zero and variance of 10^4^. Selection of covariates included in the final regression model was performed through stepwise backward selection.

To reduce bias caused by informative missingness, multiple imputation was performed, followed by Bayesian logistic regression on each imputed data set, using Gibbs sampling. Posterior distribution of the parameters of the model were then obtained by pooling of each model parameter posterior distribution sample. Convergence of Markov chains was visually assessed (Data S2).

To assess consistency of the presented results, sensitivity analyses were performed including frequentist logistic regression and Bayesian logistic regression with different priors. 95% credible intervals (95% CrI; i.e., the interval in which the parameter has a 95% probability of being) were obtained by the highest‐density method. Analyses were performed using R and JAGS software,^16^, ^17^ with rjags, mice, and HDInterval packages.^18^, ^19^, ^20^ Details on the statistical model are available in Data [Link], [Link], [Link], [Link], [Link], [Link].

## RESULTS

**FIGURE 1:**
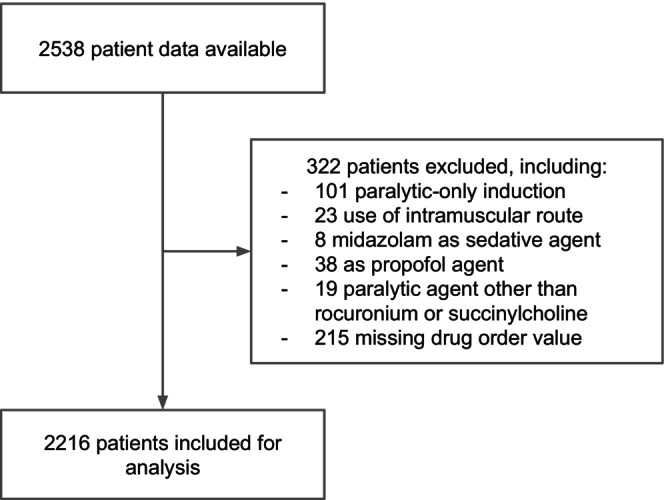
Flowchart of the study.

There were 2538 subjects included in the cohort. After application of exclusion criteria, 2216 patients were included for analysis. A flowchart is reported in Figure 1. Characteristics of the study subjects are reported in Table 1. Median (IQR) age was 47.0 (32.0–63.0) years. Medications administered for induction prior to intubation included the sedatives etomidate (1943, 88.6%) or ketamine (251, 11.4%) and the paralytics rocuronium (1699, 77.4%) or succinylcholine (495, 22.6%). A total of 1254 (56.6%) patients received rapid sequence intubation with paralytic as the first agent and 962 (43.4%) with sedative as the first agent. Overall, first‐attempt failure occurred in 110 patients (5.0%), including 54 (4.3%) with a paralytic‐first strategy and 56 (5.9%) with a sedative‐first strategy. Missing value proportions are reported in Data S5.

**TABLE 1:** Characteristics of the population.

	Paralytic first (*n* = 1254, 56.6%)	Sedative first (*n* = 962, 43.4%)	Overall (*N* = 2216)
Demographics			
Age (years)	45.0 (31.0–62.0)	49.0 (33.0–64.0)	47.0 (32.0–63.0)
Sex, female	855 (68.2)	650 (67.6)	1505 (67.9)
BMI (kg/m^2^)	26.5 (23.0–31.5)	26.8 (23.3–31.8)	26.7 (23.1–31.6)
Race/ethnicity			
American Indian or Alaskan Native	87 (6.9)	58 (6.0)	145 (6.5)
Asian	21 (1.7)	25 (2.6)	46 (2.1)
Black, non‐Hispanic	307 (24.5)	281 (29.2)	588 (26.5)
Hispanic	71 (5.7)	68 (7.1)	139 (6.3)
Native Hawaiian or Pacific	1 (0.1)	1 (0.1)	2 (0.1)
White, non‐Hispanic	449 (35.8)	321 (33.4)	770 (34.7)
Other	297 (23.7)	200 (20.8)	497 (22.4)
Patient declined to report or nonreported	21 (1.7)	8 (0.8)	29 (1.3)
Indication for intubation			
Medical	973 (78.1)	767 (80.3)	1740 (79.1)
Trauma	273 (21.9)	188 (19.7)	461 (20.9)
Vital signs at induction			
Heart rate (beats/min)	102 (82.0–121)	101 (84.0–120)	102 (83.0–120)
Systolic blood pressure (mm Hg)	117 (77.5–142)	119 (85.0–143)	118 (81.5–143)
Pulse oximetry (%)	99.0 (96.0–100)	100 (97.0–100)	99.0 (96.5–100)
Combinations of sedative and paralytic induction agents			
Etomidate–succinylcholine	213 (17.2)	223 (23.3)	436 (19.9)
Etomidate–rocuronium	941 (76.0)	566 (59.2)	1507 (68.7)
Ketamine–succinylcholine	17 (1.4)	42 (4.4)	59 (2.7)
Ketamine–rocuronium	67 (5.4)	125 (13.1)	192 (8.7)
Outcomes			
First‐attempt failure	54 (4.3)	56 (5.9)	110 (5.0)
Hypoxemia (SpO_2_ < 90%) during procedure	239 (21.6)	189 (21.6)	428 (21.6)
Complications			
Major^a^	272 (24.8)	215 (24.8)	487 (24.8)
Any^b^	281 (25.6)	223 (25.7)	504 (25.6)

*Note*: Quantitative variables are reported as median (IQR). Categorial variables are reported with absolute value and proportion (%).

Abbreviation: BMI, body mass index.

^a^ Major complication is defined by occurrence of any event among witnessed aspiration, cardiac arrest within 5 min after induction, esophageal intubation, SpO_2_ < 90% during procedure.

^b^ Any complication is defined by occurrence of a major complication or pharyngeal, laryngeal, tracheal or dental trauma or pneumothorax.

### Primary outcome

Age, sex, BMI and induction agents were included as adjustment variables in the final model (Data S2). Coefficients of the Bayesian logistic regression model are reported in Table 2.

**TABLE 2:** Bayesian logistic regression coefficients estimates and 95% highest‐density CrIs for the outcome of first‐attempt failure.

Variable	OR estimate	95% CrI
Age	1.27	(1.03–1.53)
Sex, female	0.79	(0.48–1.15)
BMI	0.95	(0.73–1.17)
Sedative agent		
Etomidate	Reference	
Ketamine	0.33	(0.08–0.64)
Paralytic agent		
Succinylcholine	Reference	
Rocuronium	0.75	(0.44–1.08)
Drug sequence order		
Sedative first	Reference	
Paralytic first	0.73	(0.46–1.02)

*Note*: ORs less than 1 indicate reduction of risk of first‐attempt failure.

Abbreviation: CrI, credible interval.

**FIGURE 2:**
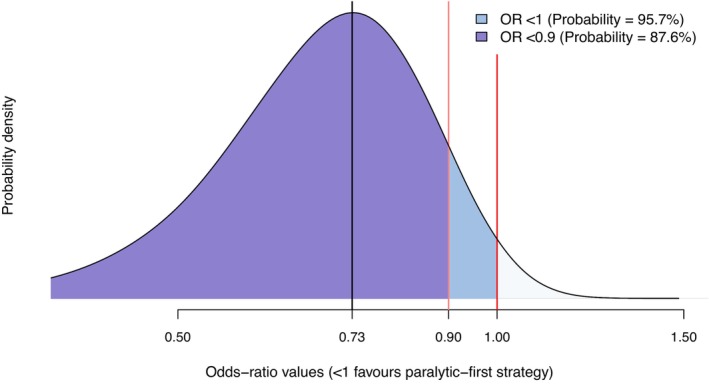
Posterior probability distribution of the OR of drug sequence order for risk of first‐attempt failure and areas under curves with integrals for OR inferior to 1 and to 0.9.

A paralytic‐first strategy was associated with reduced risk of first‐attempt failure after adjustment, with an estimated OR of 0.73 (95% CrI 0.46–1.02). The probability that the adjusted OR was less than 1 was 95.7%. The probability that the adjusted OR was less than 0.9 was 87.6%. Figure 2 describes the posterior probability density of the OR.

### Secondary outcomes

Table 3 presents the estimates of the adjusted OR of drug sequence order for other outcomes. OR estimates for risk of hypoxemia during the procedure was 0.99 (0.78–1.22), and the probability that the OR was inferior to 1 was 55.0%. Detailed results for other clinical endpoints are reported in Table 3. Stratified analysis for every complication is detailed in Data S6. Due to the low number of events for every complication, no significant tendency has been identified.

**TABLE 3:** Bayesian estimates of ORs, 95% highest‐density CrIs, and probabilities of OR < 1 and OR < 0.9 for risk of hypoxemia or occurrence of major and any complication.

Response variable	OR	95% CrI	Posterior probability		
			OR < 1	OR < 0.9	OR > 1.1
Key secondary endpoint					
Hypoxemia during procedure	0.99	(0.78–1.22)	55.0%	20.3%	17.0%
Other clinical endpoints					
Major complication^a^	1.00	(0.80–1.22)	50.9%	16.9%	18.6%
Any complication^b^	1.00	(0.79–1.22)	53.5%	19.0%	17.2%

Abbreviation: CrI, credible interval.

^a^ Major complication is defined by occurrence of any event among witnessed aspiration, cardiac arrest within 5 min after induction, esophageal intubation, or SpO_2_ < 90% during procedure.

^b^ Any complication is defined by occurrence of a major complication or pharyngeal, laryngeal, tracheal or dental trauma, or pneumothorax.

### Exploratory analyses

Table 4 reports the proportion of first‐attempt failure depending on the sedative and paralytic agents and drug sequence order. In patients receiving etomidate, paralytic‐first subgroups showed lower first‐attempt failure rates than sedative‐first subgroups, whatever the paralytic agent used. Subgroups for patients receiving ketamine were too small to make comparisons.

**TABLE 4:** Observed prevalence of first‐attempt failure among induction agent combinations subgroups on nonimputed data without missing values on sedative, paralytic agents, and drug sequence order.

	Sedative agent		
	Etomidate (*n* = 1897)	Ketamine (*n* = 238)	Overall (*N* = 2135)
Paralytic agent			
Succinylcholine (*n* = 474)			
Paralytic first	10/205 (4.9%)	2/16 (12.5%)	12/221 (5.4%)
Sedative first	17/213 (8.0%)	1/40 (2.5%)	18/253 (7.1%)
Overall	27/418 (6.5%)	3/56 (5.4%)	30/474 (6.3%)
Rocuronium (*n* = 1661)			
Paralytic first	42/922 (4.6%)	0/65 (0.0%)	42/987 (4.3%)
Sedative first	34/557 (6.1%)	2/117 (1.7%)	36/674 (5.3%)
Overall	76/1479 (5.1%)	2/182 (1.1%)	78/1661 (4.7%)
Overall (*n* = 2135)			
Paralytic first	52/1127 (4.6%)	2/81 (2.5%)	54/1208 (4.5%)
Sedative first	51/770 (6.6%)	3/157 (1.9%)	54/927 (5.8%)
Overall	103/1897 (5.4%)	5/238 (2.1%)	108/2135 (5.1%)

### Sensitivity analyses

Results of sensitivity analyses are available in Data S3 and S4. Both the frequentist logistic regression model and the Bayesian logistic regression models with varying priors resulted in estimates consistent with the primary analyses.

## DISCUSSION

In this retrospective cohort analysis, we investigated the effect of the sequence of administration of induction drugs on the likelihood of tracheal intubation first‐attempt failure. Our findings indicate that a paralytic‐first strategy could be associated with a significantly lower likelihood of first‐attempt failure. No effect was identified on occurrence of complications nor periprocedure hypoxemia, which could result from the performance of preventive measures such as quality preoxygenation and use of adapted intubation devices and protocols, which enable adapted action from the operator before occurrence of complication. No major heterogeneity of this effect was observed across various drug combinations, albeit with limited interpretability in certain subgroups due to small sample sizes.

These results differ from previously published results describing performance and safety of paralytic‐first sequence (named “timing principle”) in the operating theater. Several small‐sample‐size studies conducted in preoperative settings indicated similar intubation conditions between paralytic‐first and sedative‐first strategies.^9^, ^10^, ^21^, ^22^ However, none of these studies directly compared first‐attempt failure rates, and the sample sizes were possibly too small to identify any difference on this criterion. Also, the drugs used both as sedative (mostly thiopental and propofol) and paralytic (vecuronium, atracurium) differ from our study, with a different pharmacokinetic profile.

In the ED, our findings differ with results of Driver et al.,^6^ who reported no significant difference in first‐attempt failure rates between sedative‐first and paralytic‐first strategies on unadjusted analysis. However, our study benefits from a larger sample size and adjustment for potential confounding factors, which may account for the discrepancy.

Driver et al. noted a shorter procedure duration with the paralytic‐first approach when the paralytic was administered first. This delay reduction between loss of spontaneous ventilation and adequate sedation could participate in the explanation of our results. Indeed, this effect is consistent with the pharmacokinetic concept supporting the potential superiority of a paralytic‐first strategy compared to sedative‐first, particularly if the delay between sedative and paralytic administration is increased.

## LIMITATIONS

Several limitations should be acknowledged. First, the retrospective nature of our study introduces inherent biases in data collection and patient selection. However, the data collection process was prospective and systematic, and the design of our study was performed posterior to data collection, excluding the risk of experimentator information bias.

Second, the final model was adjusted on age, BMI, sex, and drug combination used, although no major difference between the groups was identified with regard to other measured factors, unmeasured factors such as other comorbidities, variations in clinical practice, or operator experience could influence our findings.

Third, the Bayesian approach used in our analysis relies on specific assumptions. In particular, the choice of prior distribution could theoretically affect posterior results. In this study, we privileged priors with almost equal likelihood on a wide parameter space. Some authors advocate for use of less flat distributions, to regularize the extreme inferences that can be obtained from maximum likelihood or completely noninformative priors.^23^ However, sensitivity analyses yielded consistent results with both different prior distributions and frequentist analysis, which advocate for robustness of these results.

Fourth, the criterion of first‐attempt failure, while commonly used as an outcome measure, may not directly correlate with clinical outcomes. Other factors such as rates of desaturation, trauma to airway structures, and long‐term patient outcomes should be considered in future studies to provide a more comprehensive assessment of procedural success and patient safety.

Fifth, the absence of data on awake paralysis limits our ability to assess its potential impact on our findings. Awake paralysis may lead to adverse patient experience and later outcomes such as the development of posttraumatic stress disorder, so it should be considered in future studies. However, the hypothetical risk of awake paralysis during paralytic‐first induction has not been supported by experimental data either in the operating theater^9^, ^10^, ^22^ or in the ED.^8^ Based on these results, a paralytic‐first strategy should be considered as a safe practice.

## CONCLUSIONS

In this analysis of a large single‐center prospective cohort of patients undergoing emergency tracheal intubation, a paralytic‐first induction strategy was associated with a reduced risk of first‐attempt failure compared to a sedative‐first approach.

## AUTHOR CONTRIBUTIONS

All authors conceived the study. Brian Driver and Matthew E. Prekker collected and prepared the data. Pierre Catoire performed the statistical analysis. All authors wrote and approved the final manuscript. All authors have contributed to significant changes to the manuscript. All authors are responsible for the final version of the manuscript.

## CONFLICT OF INTEREST STATEMENT

The authors declare no conflicts of interest.

## Supporting information


**Data S1.** Additional file 1. Bayesian model used for analysis.

**Data S2.** Additional file 2. Convergence of Monte‐Carlo Markov Chains.

**Data S3.** Additional file 3: result of sensitivity analyses—frequentist analysis.

**Data S4.** Additional file 4: result of sensitivity analyses—different priors.

**Data S5.** Additional file 5: missing values proportions.

**Data S6.** Additional file 6: stratified Bayesian regression for every complication.

## Data Availability

The data sets analyzed during the current study are available from BD and MEP on reasonable request.
