# Supplementary figures and images for: Effect of administration sequence of induction agents on first‐attempt failure during emergency intubation: A Bayesian analysis of a prospective cohort
Source: Acad Emerg Med. 2024 Oct 18;32(2):123–9. doi: 10.1111/acem.15031 (PMC11816003; doi:10.1111/acem.15031)

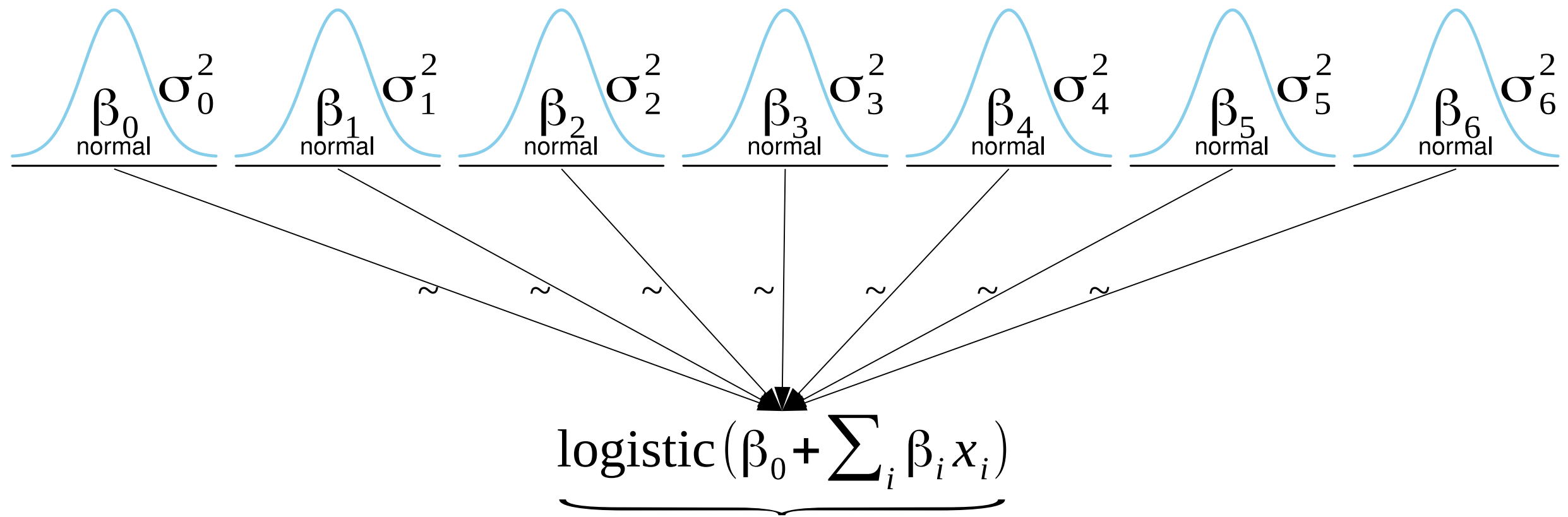

Prior distribution parameters for main analysis:

$$\beta_i = 0$$

$$\sigma_i^2 = 1000$$

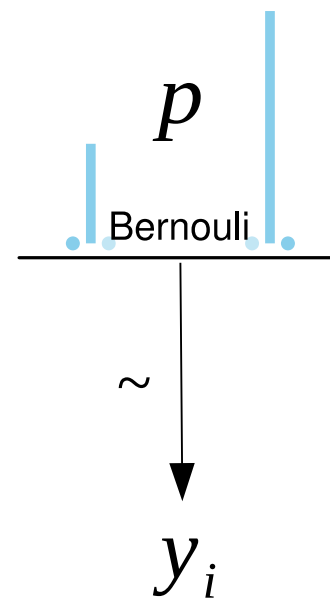

Supplement: Supplementary file 1 — Data S1. Additional file 1. Bayesian model used for analysis. [file ACEM-32-123-s005.pdf]
