# Supplementary material for: Effect of administration sequence of induction agents on first‐attempt failure during emergency intubation: A Bayesian analysis of a prospective cohort
Source: Acad Emerg Med. 2024 Oct 18;32(2):123–9. doi: 10.1111/acem.15031 (PMC11816003; doi:10.1111/acem.15031)
Supplement: Supplementary file 2 — Data S2. Additional file 2. Convergence of Monte‐Carlo Markov Chains. [file ACEM-32-123-s006.pdf]

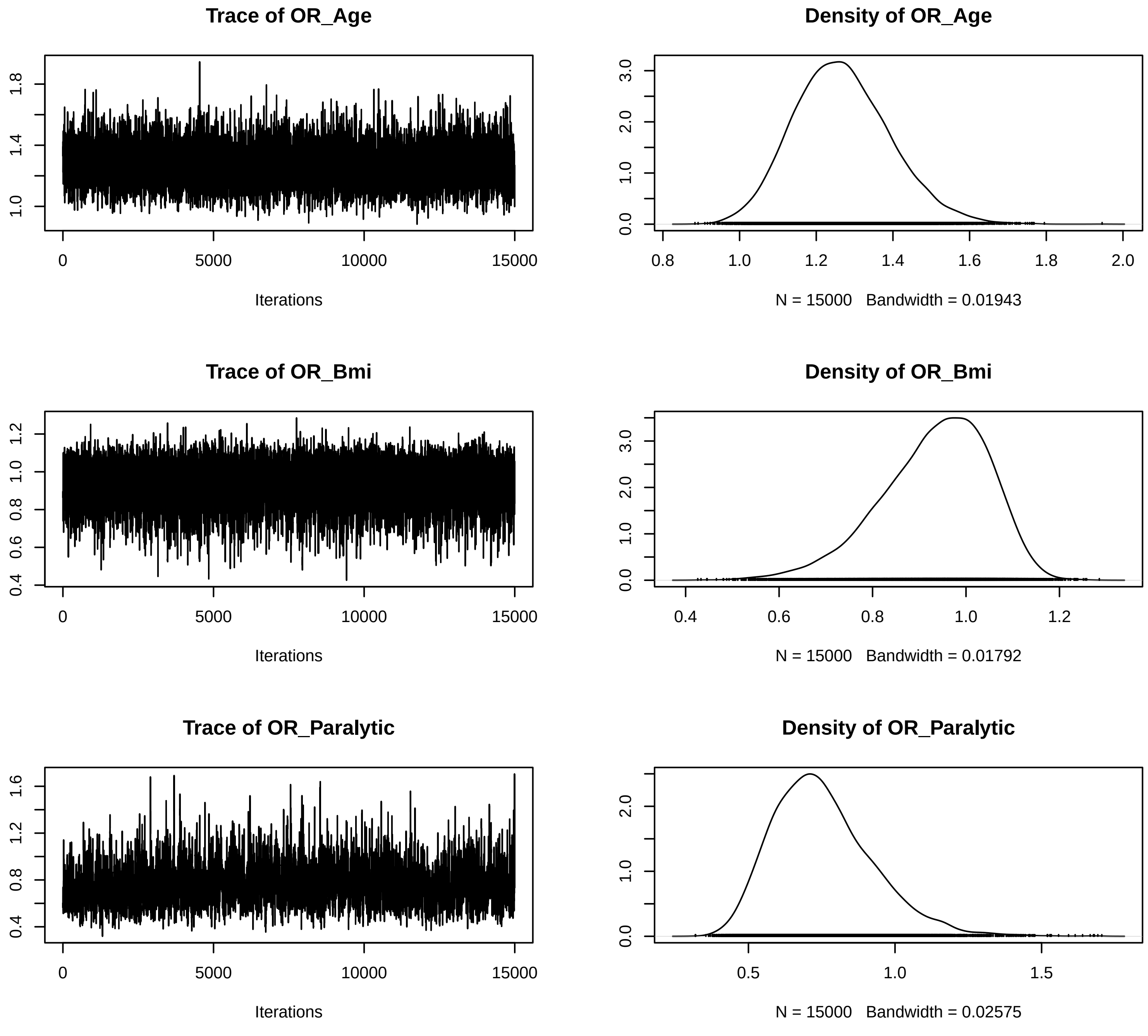
Trace of OR_Age
Iterations
Density of OR_Age
N = 15000 Bandwidth = 0.01943
Trace of OR_Bmi
Iterations
Density of OR_Bmi
N = 15000 Bandwidth = 0.01792
Trace of OR_Paralytic
Iterations
Density of OR_Paralytic
N = 15000 Bandwidth = 0.02575

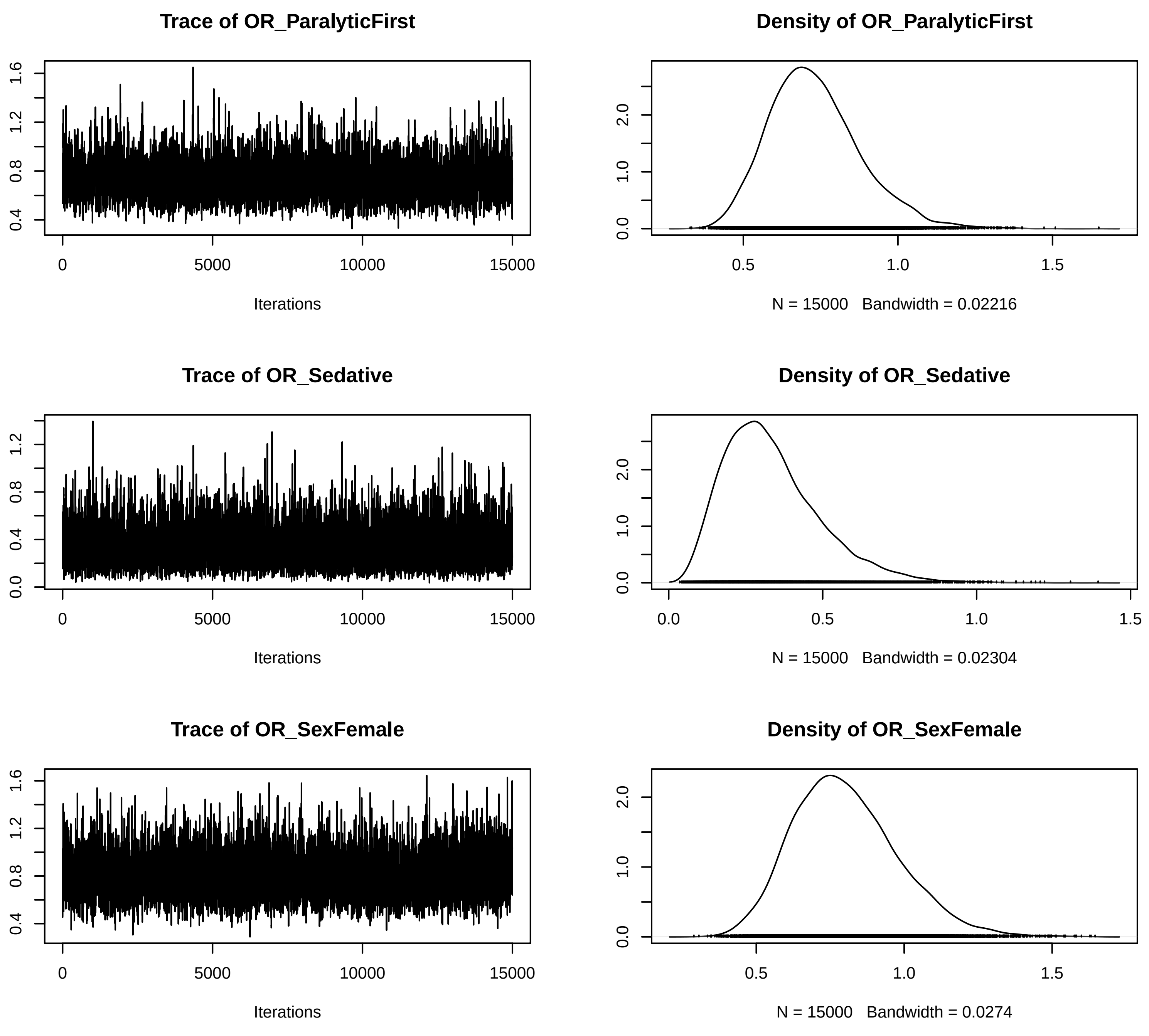
Trace of OR_ParalyticFirst
Iterations
Density of OR_ParalyticFirst
N = 15000 Bandwidth = 0.02216
Trace of OR_Sedative
Iterations
Density of OR_Sedative
N = 15000 Bandwidth = 0.02304
Trace of OR_SexFemale
Iterations
Density of OR_SexFemale
N = 15000 Bandwidth = 0.0274
