# Supplementary material for: Effect of administration sequence of induction agents on first‐attempt failure during emergency intubation: A Bayesian analysis of a prospective cohort
Source: Acad Emerg Med. 2024 Oct 18;32(2):123–9. doi: 10.1111/acem.15031 (PMC11816003; doi:10.1111/acem.15031)
Supplement: Supplementary file 3 — Data S3. Additional file 3: result of sensitivity analyses—frequentist analysis. [file ACEM-32-123-s004.docx]

**Additional file 4 - frequentist logistic regression analysis on imputed data**

Model: $FirstPassFailure\sim DrugOrder+Age+Sex+BMI+Sedative+Paralytic$

| **Variable** | **OR estimate** | **95% confidence interval** | **p-value** |
| --- | --- | --- | --- |
| Age | 1.26 | [1.04-1.53] | < 0.05 |
| Sex, female | 0.79 | [0.51-1.21] | 0.27 |
| Body mass index | 0.99 | [0.81-1.22] | 0.94 |
| Sedative agent |  |  |  |
| - Etomidate | Reference |  |  |
| - Ketamine | 0.32 | [0.13-0.81] | < 0.05 |
| Paralytic agent |  |  |  |
| - Succinylcholine | Reference |  |  |
| - Rocuronium | 0.72 | [0.47-1.11] | 0.14 |
| Drug sequence order |  |  |  |
| - Sedative-first | Reference |  |  |
| - Paralytic-first | 0.72 | [0.44-1.12] | 0.10 |
