# Supplementary material for: Effect of administration sequence of induction agents on first‐attempt failure during emergency intubation: A Bayesian analysis of a prospective cohort
Source: Acad Emerg Med. 2024 Oct 18;32(2):123–9. doi: 10.1111/acem.15031 (PMC11816003; doi:10.1111/acem.15031)
Supplement: Supplementary file 4 — Data S4. Additional file 4: result of sensitivity analyses—different priors. [file ACEM-32-123-s003.docx]

**Additional file 5 - bayesian logistic regression analysis on imputed data**

Model: $FirstPassFailure\sim DrugOrder+Age+Sex+BMI+Sedative+Paralytic$

Priors: $\beta\sim N(\mu=0,\sigma^{2}=1)$

| **Variable** | **OR estimate** | **95% credible interval** |
| --- | --- | --- |
| Age | 1.29 | [1.03-1.55] |
| Sex, female | 0.81 | [0.47-1.17] |
| Body mass index | 0.94 | [0.70-1.13] |
| Sedative agent |  |  |
| - Etomidate | Reference |  |
| - Ketamine | 0.37 | [0.08-0.69] |
| Paralytic agent |  |  |
| - Succinylcholine | Reference |  |
| - Rocuronium | 0.80 | [0.47-1.16] |
| Drug sequence order |  |  |
| - Sedative-first | Reference |  |
| - Paralytic-first | 0.74 | [0.47-1.04] |

Priors: $\beta\sim N(\mu=0,\sigma^{2}=10)$

| **Variable** | **OR estimate** | **95% credible interval** |
| --- | --- | --- |
| Age | 1.29 | [1.04-1.55] |
| Sex, female | 0.82 | [0.50-1.20] |
| Body mass index | 0.94 | [0.71-1.14] |
| Sedative agent |  |  |
| - Etomidate | Reference |  |
| - Ketamine | 0.36 | [0.09-0.69] |
| Paralytic agent |  |  |
| - Succinylcholine | Reference |  |
| - Rocuronium | 0.79 | [0.47-1.17] |
| Drug sequence order |  |  |
| - Sedative-first | Reference |  |
| - Paralytic-first | 0.75 | [0.46-1.05] |

Priors: $\beta\sim N(\mu=0,\sigma^{2}=1000000)$

| **Variable** | **OR estimate** | **95% credible interval** |
| --- | --- | --- |
| Age | 1.30 | [1.04-1.56] |
| Sex, female | 0.82 | [0.59-1.18] |
| Body mass index | 0.94 | [0.71-1.15] |
| Sedative agent |  |  |
| - Etomidate | Reference |  |
| - Ketamine | 0.35 | [0.08-0.68] |
| Paralytic agent |  |  |
| - Succinylcholine | Reference |  |
| - Rocuronium | 0.80 | [0.46-1.16] |
| Drug sequence order |  |  |
| - Sedative-first | Reference |  |
| - Paralytic-first | 0.74 | [0.48-1.05] |
