# Supplementary material for: Effect of administration sequence of induction agents on first‐attempt failure during emergency intubation: A Bayesian analysis of a prospective cohort
Source: Acad Emerg Med. 2024 Oct 18;32(2):123–9. doi: 10.1111/acem.15031 (PMC11816003; doi:10.1111/acem.15031)
Supplement: Supplementary file 5 — Data S5. Additional file 5: missing values proportions. [file ACEM-32-123-s002.docx]

Missing values proportions for the population characteristics, including patients with missing value for drug administration sequence (N = 147)

|  | **Paralytic first**  **(N=1254)** | **Sedative first**  **(N=962)** | **Missing sequence**  **(N=147)** | **Overall**  **(N=2363)** |
| --- | --- | --- | --- | --- |
| **Demographics** |  |  |  |  |
| ***Age (years)*** | 45.0 [31.0, 62.0] | 49.0 [33.0, 64.0] | 49.5 [30.0, 63.0] | 48.0 [32.0, 63.0] |
| Missing | 0 (0%) | 1 (0.1%) | 49 (33.3%) | 50 (2.1%) |
| ***Sex, female*** | 855 (68.2%) | 650 (67.6%) | 71 (48.3%) | 1576 (66.7%) |
| Missing | 0 (0%) | 0 (0%) | 49 (33.3%) | 49 (2.1%) |
| ***Body Mass Index (kg/m²)*** | 26.5 [23.0, 31.5] | 26.8 [23.3, 31.9] | 26.7 [23.7, 29.8] | 26.7 [23.1, 31.6] |
| Missing | 101 (8.1%) | 71 (7.4%) | 77 (52.4%) | 249 (10.5%) |
| ***Ethnicity*** |  |  |  |  |
| Non-hispanic or latino | 872 (69.5%) | 691 (71.8%) | 50 (34.0%) | 1613 (68.3%) |
| Hispanic or latino | 71 (5.7%) | 68 (7.1%) | 7 (4.8%) | 146 (6.2%) |
| Unknown | 306 (24.4%) | 201 (20.9%) | 41 (27.9%) | 548 (23.2%) |
| Missing | 5 (0.4%) | 2 (0.2%) | 49 (33.3%) | 56 (2.4%) |
| **Indication for intubation** |  |  |  |  |
| Medical | 973 (77.6%) | 767 (79.7%) | 66 (44.9%) | 1806 (76.4%) |
| Trauma | 273 (21.8%) | 188 (19.5%) | 23 (15.6%) | 484 (20.5%) |
| Missing | 8 (0.6%) | 7 (0.7%) | 58 (39.5%) | 73 (3.1%) |
| **Vital signs at induction** | | | | |
| ***Heart Rate (bpm)*** | 102 [82.0, 121] | 101 [84.0, 120] | 113 [92.3, 125] | 101 [81.0, 120] |
| Missing | 934 (74.5%) | 489 (50.8%) | 133 (90.5%) | 1522 (64.4%) |
| ***Systolic Blood Pressure (mmHg)*** | 117 [77.5, 142] | 119 [85.0, 143] | 134 [107, 156] | 115 [-9.00, 142] |
| Missing | 935 (74.6%) | 490 (50.9%) | 138 (93.9%) | 1524 (64.5%) |
| ***SpO2 (%)*** | 99.0 [96.0, 100] | 100 [97.0, 100] | 99.5 [96.8, 100] | 99.0 [96.0, 100] |
| Missing | 934 (74.5%) | 487 (50.6%) | 139 (94.6%) | 1521 (64.4%) |
| **Induction agents** |  |  |  |  |
| Etomidate - succinylcholine | 213 (17.0%) | 223 (23.2%) | 10 (6.8%) | 446 (18.9%) |
| Etomidate - rocuronium | 941 (75.0%) | 566 (58.8%) | 24 (16.3%) | 1531 (64.8%) |
| Ketamine - succinylcholine | 17 (1.4%) | 42 (4.4%) | 1 (0.7%) | 60 (2.5%) |
| Ketamine - rocuronium | 67 (5.3%) | 125 (13.0%) | 3 (2.0%) | 195 (8.3%) |
| Missing | 16 (1.3%) | 6 (0.6%) | 109 (74.1%) | 131 (5.5%) |
| **Outcomes** |  |  |  |  |
| ***Hypoxemia*** | 239 (19.1%) | 189 (19.6%) | 12 (8.2%) | 440 (18.6%) |
| Missing | 147 (11.7%) | 88 (9.1%) | 110 (74.8%) | 345 (14.6%) |
| ***First pass failure*** | 54 (4.3%) | 56 (5.8%) | 7 (4.8%) | 117 (5.0%) |
| Missing | 11 (0.9%) | 9 (0.9%) | 50 (34.0%) | 70 (3.0%) |
| ***Complications - major*** | 272 (21.7%) | 215 (22.3%) | 16 (10.9%) | 503 (21.3%) |
| Missing | 156 (12.4%) | 95 (9.9%) | 110 (74.8%) | 361 (15.3%) |
| ***Complications - all*** | 281 (22.4%) | 223 (23.2%) | 16 (10.9%) | 520 (22.0%) |
| Missing | 156 (12.4%) | 95 (9.9%) | 110 (74.8%) | 361 (15.3%) |
