# Supplementary material for: Effect of administration sequence of induction agents on first‐attempt failure during emergency intubation: A Bayesian analysis of a prospective cohort
Source: Acad Emerg Med. 2024 Oct 18;32(2):123–9. doi: 10.1111/acem.15031 (PMC11816003; doi:10.1111/acem.15031)
Supplement: Supplementary file 6 — Data S6. Additional file 6: stratified Bayesian regression for every complication. [file ACEM-32-123-s001.docx]

Estimates of odd-ratios for paralytic-first sequence on individual complications, adjusted on age, sex, BMI, sedative and paralytic agents

| **Response variable** | **OR** | **95% CrI** | **Posterior probability** | | |
| --- | --- | --- | --- | --- | --- |
|  |  |  | **OR < 1** | **OR < 0.9** | **OR > 1.1** |
| **Major complications** | 1.00 | [0.80-1.22] | 50.9% | 16.9% | 18.6% |
| Cardiac arrest within 5 minutes after induction | 1.44 | [0.14-3.69] | 45.1% | 39.3% | 49.4% |
| Hypoxemia during procedure | 0.99 | [0.78-1.22] | 55.0% | 20.3% | 17.0% |
| Esophageal intubation | No convergence* | | | | |
| Aspiration | 0.86 | [0.16-1.82] | 71.6% | 64.6% | 22.9% |
| **Other complications** |  |  |  |  |  |
| Pharyngeal trauma | 1.96 | [0.03-5.68] | 37.2% | 32.9% | 58.5% |
| Laryngeal trauma | 3.83 | [0.24-10.44] | 9.7% | 7.8% | 87.8% |
| Tracheal trauma | 1.48 | [0.15-3.63] | 40.9% | 34.8% | 53.4% |
| Dental trauma | 0.43 | [0.01-1.14] | 92.3% | 90.7% | 5.6% |
| Pneumothorax | No convergence* | | | | |
| **All complications** | 1.00 | [0.79-1.22] | 53.5% | 19.0% | 17.2% |
| *Regressions on esophageal intubation and pneumothorax did not converge due to low number of events (N=3 and 2 respectively). | | | | | |
